# Treatment Utilization Pattern of Preschool Children With Attention-Deficit/Hyperactivity Disorder

**DOI:** 10.1177/10870547231215287

**Published:** 2023-12-12

**Authors:** Raman Baweja, Ritika Baweja, Hunter Weidlich, Jennifer E. Nyland, Daniel A. Waschbusch, James G. Waxmonsky

**Affiliations:** 1 Pennsylvania State College of Medicine, Hershey, PA, USA

**Keywords:** preschoolers, ADHD, CNS stimulants, alpha-2 agonists, therapy

## Abstract

**Objective:**

The aim of this study was to identify patterns of ADHD care, including factors that guide selection and sequencing of treatments in a large nationwide sample of preschool-aged youth over the past 6 years.

**Method:**

A retrospective cohort study utilizing a large electronic health record (TriNetX) of nearly 24,000 children ages 3 to 6 diagnosed with ADHD.

**Results:**

One in three preschoolers with ADHD were prescribed psychotropic medication, most commonly methylphenidate and guanfacine. One in 10 had at least one psychotherapy billing code during the entire assessment with most youth starting medication before psychotherapy. Rates of most treatments, including polypharmacy, increased with comorbid psychiatric disorders or sleep problems and over the course of the coronavirus pandemic.

**Conclusion:**

Rates of treatment have increased over time but are still largely inconsistent with published care guidelines that advise therapy before medication. Clinicians appear to prioritize psychiatric comorbidity and sleep problems when selecting treatments.

**Tribute to Joe Biederman (By Dr. James G. Waxmonsky)**

I worked as a child fellow at MGH with Dr. Biederman and then he offered me my first job outside of fellowship. I have two core memories of him. The first was him asking me about the frequency of depression in elementary school aged boys versus girls. I was proud of myself for knowing the answer, but he immediately asked me why the rates differed. There I was stuck. This was just one example of his efforts to push me past the what and onto the why. If our field didn’t have an answer for the why, then you had a new research idea. This constant generation of academic inquisitiveness drawn from clinical experience is what I try to embody today, and Dr. Biederman was one of my mentors that inspired this drive.

The second core memory was being asked to cover his patients while he was away. I quickly discovered that his families knew how to reach him directly and that he responded personally to parents and patients whether it was about a child from Charlestown with Mass Medicaid or a literal prince from across the globe. That experience has always grounded me in the reality that all academic efforts are ultimately about helping patients and families and that care needs to be accessible. Despite whatever the demands of my schedule, every day I channel my inner Joe and find the time to personally speak with patients and parents.

## Introduction

ADHD affects ∼5% to 11% of children and adolescents, with a reported 2% to 2.5% of preschoolers meeting the diagnostic criteria of the disorder (Danielson, Bitsko et al., 2018; Wolraich et al., 2019). The prevalence of ADHD among children, including those under age 6, has increased 1.5-fold from 1997 to 2016 (Xu et al., 2018) and the percentage of preschoolers who are receiving clinical care for ADHD has nearly doubled in the last decade (Visser et al., 2016). ADHD in preschool age youth is associated with a range of challenges including behavioral challenges at home, motor coordination problems and impairments in peer relationships (DuPaul et al., 2001). School functioning is often impaired by ADHD (Baweja et al., 2015) and preschoolers with ADHD symptoms often show reduced school readiness, which predicts poor academic achievement in late elementary school (Perrin et al., 2019). Even when diagnosed in the preschool years, the disorder often persists into middle childhood. It is an established risk factor for later serious psychopathology and emphasizes the importance of timely treatment.

According to revised clinical practice guidelines released by the American Academy of Pediatrics (AAP) in 2019 and the European National Institute of Healthcare and Excellence, clinicians should prescribe evidence-based behavioral interventions as the first line of treatment to preschoolers with ADHD (National Institute for Health and Clinical Evidence [NICE], 2018; Wolraich et al., 2019). If there is moderate-to-severe impairment and if non-pharmacological interventions do not provide significant improvement, methylphenidate (MPH) is recommended (NICE, 2018; Wolraich et al., 2019). While the efficacy of behavioral interventions for improving symptoms of ADHD is less than that seen with medications, multiple studies have found significant effects of behavioral therapies for improving an array of conduct problems as well as parenting practices in preschool age youth with ADHD (Daley et al., 2014; Groenman et al., 2022; Halperin & Marks, 2019; Rimestad et al., 2019). Despite their established efficacy, several studies have reported concerningly low rates of behavioral therapy used in unmedicated and medicated youth with ADHD (Halperin & Marks, 2019). For example, children aged 2 to 5 years receiving treatment for ADHD were more likely to receive pharmacological treatment than psychological services (Visser et al., 2016; Waxmonsky et al., 2019). Even in areas where counseling resources are plentiful, utilization is low (Gellad et al., 2014). Due to this low rate of therapy uptake, only about 16% of medicaid-enrolled preschool age children with ADHD received care during 2005 to 2012 that was consistent with recommended care guidelines (Moran et al., 2019).

Rates of ADHD medication usage amongst children and young adults in the United States (U.S.) nearly doubled between 2006 and 2015 (Girand et al., 2020). Recent evidence shows that prescription of stimulants for children ages 5 to 9 has decreased slightly from 2016 to 2021 (Danielson, Bohm et al., 2023). Much less is known about recent medication patterns in children under 5. Receipt of multiple ADHD medications (polypharmacy), increased from 16.8% to 20.5%, whereas use of any two psychotropics in children prescribed at least one ADHD medication increased from 26.0% to 40.7%. The most common combination of ADHD medications was CNS stimulants and α-2 agonists (67.1%). Nearly one in seven (14.4%) youth prescribed ADHD medication were also taking a selective serotonin reuptake inhibitor (SSRIs) (Girand et al., 2020). Similar increases have been reported in other countries over the early part of this century (Brault & Lacourse, 2012). Despite apparent increases in use of second-generation antipsychotics (SGAs) among adolescents and young adults, prescriptions for SGAs among young children (ages 1–6 years) remained low and stable between 2006 and 2010 (0.14% and 0.11%, respectively). However, the most common diagnosis for those treated with SGAs was ADHD (52.5%), with 11.8% of preschoolers taking an ADHD medication also prescribed an SGAs (Brault & Lacourse, 2012).

In this age range, there is a dearth of published information about why some prescribers select certain treatments over others or start one before or another, which may contribute to practice patterns that are inconsistent with care guidelines. It has been theorized that psychiatric and medical comorbidities may drive prescriber preference; however, this topic has not been well examined to date (Baweja & Waxmonsky, 2018, 2022; Faraone, 2018; Girand et al., 2020). Prior research on treatment patterns in preschool aged children with ADHD has been limited in scope and reach, with studies limited to a specific region or prescriber type (Girand et al., 2020; Harstad et al., 2021; Klein et al., 2016). Improving the understanding of what drives treatment preferences is the first step toward improving uptake of evidenced-based interventions (e.g., parent and classroom based behavioral therapies) and reducing rates of treatments with less established efficacy profiles or more concerning tolerability profiles (e.g., SGAs).

There is limited data on treatment trends over the past 5 years, so little is known about the impact of the coronavirus pandemic on psychotropic medication use in preschoolers. Across all ages, there has been an appreciable increase in the percent of the population using ADHD medications versus rates seen prior to the pandemic (Gimbach et al., 2023). In addition to ADHD medications, use of most other psychotropics has increased considerably over the pandemic (Bliddal et al., 2023; Gimbach et al., 2023; Leong et al., 2022) but patterns have not been examined specifically in preschool aged youth with ADHD. The aim of this study was to identify ADHD treatment patterns in a large nationwide sample of preschool aged youth over the past 6 years including examination of factors that may guide providers selection and sequencing of treatments.

## Methods

**Study design:** This study was conducted with data obtained from TriNetX, LLC (“TriNetX”), a global federated health research network that provides access to electronic health records (EHRs), including diagnoses, procedures, medications, laboratory values, and genomic information from healthcare organizations (HCOs) worldwide. The data used in this study was collected on August 31, 2023, from the TriNetX Research Network, which provided access to EHRs from approximately 110 million patients from 78 HCOs. The analysis applied a retrospective observational design utilizing EHRs from approximately 35 million patients from 78 healthcare organizations sourced from the TriNetX research network database in the U.S. (Cambridge, MA). *The data reviewed is a secondary analysis of existing data and is de-identified per the de-identification standard defined in Section §164.514(a) of the HIPAA Privacy Rule.* For these same reasons, the Institutional Review Board determined this study exempt from review, and patient consent was not required.

**Study population:** The population consisted of pediatric patients ages 3 to 6 years with a diagnosis code for ADHD (F90) between January 1, 2017, and August 31, 2023. Data collected included patient demographics (age, sex, race/ethnicity), comorbid psychiatric disorders and medical conditions, behavioral health service utilization, and prescription information as documented in each patient’s EHRs. The Appendix provides detailed information about the diagnostic codes used. Medication and psychotherapy services and procedures were identified using Current Procedural Terminology (CPT) codes. The data included body mass index (BMI), and the presence of seizure disorders because these are relevant to children’s psychotropic medication use.

**Data analysis:** All analyses were generated using the TriNetX platform software (TriNetX, Cambridge, MA) in August 2023. Descriptive analyses were used to explore cohort demographic factors and distributions of variables (medication prescription and psychotherapy). We did not include medications (e.g., diphenhydramine or melatonin) which are available over the counter in the U.S. in this analysis. We created the variable “ADHD medications” which included CNS stimulants, alpha-2 agonists, atomoxetine, viloxazine, and bupropion. Polypharmacy was defined as being on “2 medications” if children with ADHD were prescribed CNS stimulants and one another medication (alpha-2 agonists or antidepressants or antipsychotics) during the study period and on “3 medications” if they were prescribed CNS stimulants and alpha-2 agonists with one another medication (antidepressants or antipsychotics). We examined the frequency of psychotherapy and medication billing codes for patients who have respectively at least 4 and 6 months of assessment periods as a proxy for treatment persistence.

We examined the prescription patterns in relation to ethnicity and race in children with ADHD. To test whether prescription patterns differed as a function of demographic factors, we compared Hispanic children versus non-Hispanic, as well as white children versus non-white. We also examined prescription patterns concerning comorbid psychiatric disorders (autism spectrum disorder [ASD], anxiety disorders, disruptive behavior disorders, and sleep disorders) in children with ADHD. To test whether prescription patterns differed as a function of comorbid psychiatric disorders, a comparison was made between children with ADHD who also had comorbid psychiatric disorders versus those without comorbid psychiatric disorders, as well as between children with ADHD who also had sleep disorders versus those without sleep disorders. We also examined prescription patterns concerning comorbid medical conditions that could potentially impact prescribing (seizure disorder or BMI below the fifth percentile for their age) in children with ADHD and be feasibly tracked in the database. To test this, a comparison was made between children with ADHD who also had comorbid seizure disorder or low BMI versus those without comorbid medical conditions.

We examined if treatment utilization patterns were altered during the COVID-19 pandemic by creating three temporal subgroups: pre-pandemic (Jan 2017–Feb 2020), pandemic (March 2020–July 2021, acute phase corresponding to lockdowns, school/daycare closings, and remote learning), and post-pandemic (August 2021–August 2023). For this sub-analysis examining change over the course of the pandemic, we created the variable “nonstimulants” which included alpha-2 agonists and atomoxetine to examine changes in nonstimulant versus stimulant medication prescribing over time. Contrast analysis was performed to calculate odds ratios (OR) and 95% confidence intervals (CI).

## Results

### Demographic Characteristics

We identified 24,151 children with ADHD ages 3 to 6 years in the TriNetX Research Network database from January 1, 2017, to August 31, 2023. Table 1 describes the mean age, distribution of sex, race, and ethnicity of these children. Table 1 also describes the common psychiatric comorbid conditions among children with a diagnostic code for ADHD and the medical conditions relevant to ADHD medication treatment within the specified period. Specific speech and language developmental disorders constituted the most frequent comorbid conditions (42.9%). The next most common comorbid conditions included sleep disorders (25.8%), autism spectrum disorder (24.3%), disruptive behavior disorders (16.8%), and anxiety disorder unspecified (7.9%). Around 3% had a BMI below the fifth percentile for their age and 4% had seizure disorder. Treatments included prescriptions for psychotropic medications, psychotherapy services, and a combination of both (Table 2).

**Table 1.** Sociodemographic and Clinical Characteristics.

	ADHD 24,151 (%)
*Sociodemographic*	
Age at Index (SD) in years	4.13 ± 1.19
Gender	
Male	17,971 (74.41)
Female	6,138 (25.41)
Unknown	42 (0.17)
Ethnicity	
Not Hispanic or Latino	15,274 (63.24)
Hispanic or Latino	2,982 (12.34)
Unknown Ethnicity	5,895 (24.41)
Race	
White	12,902 (53.42)
Black or African American	4,590 (19.01)
Asian	365 (1.51)
American Indian and Alaska Native	90 (0.37)
Native Hawaiian or other Pacific Islander	46 (0.19
Unknown	6,158 (25.50)
*Psychiatric comorbidity*	
Autism spectrum disorder	5,871 (24.31)
Disruptive behavior disorders	4,054 (16.79)
Oppositional defiant disorder	1,401 (5.80)
Conduct disorder, childhood-onset	148 (0.61)
Disruptive mood dysregulation disorder	207 (0.86)
Depressive episode (Including MDD)	128 (0.53)
Bipolar disorder	27 (0.11)
Anxiety disorder, unspecified	1,897 (7.85)
Generalized anxiety disorder	367 (1.52)
Separation anxiety disorder of childhood	308 (1.28)
Post-traumatic stress disorder	262 (1.08)
Intellectual disabilities	341 (1.41)
Tic disorders	308 (1.28)
Sleep disorders	6,227 (25.78)
Eating disorders	750 (3.11)
Specific developmental disorders of speech and language	1,0353 (42.86)
*Other conditions*	
BMI pediatric, < or = to 5 percentiles for age	686 (2.84)
Epilepsy and recurrent seizures	964 (3.99)

*Note*. BMI = Body Mass Index; MDD = Major Depressive Disorder.

**Table 2.** Treatment Characteristic.

	24,151 (%)
ADHD medication	8,953 (37.06)
CNS stimulants	6,353 (26.31)
Methylphenidate group	5,142 (21.19)
Amphetamines group	2,462 (10.19)
Alpha-2 agonists	5,379 (22.17)
Guanfacine	3,766 (15.59)
Clonidine	2,591 (10.72)
Others	
Atomoxetine	206 (0.85)
Viloxazine	53 (0.22)
Bupropion	12 (0.05)
Antidepressants	941 (3.90)
Sertraline	323 (1.34)
Fluoxetine	247 (1.02)
Escitalopram	91 (0.38)
Trazodone	170 (0.70)
Mood stabilizers	
Valproate	185 (0.77)
Antipsychotics	709 (2.94)
Risperidone	531 (2.20)
Aripiprazole	176 (0.73)
Olanzapine	46 (0.19)
Quetiapine	40 (0.17)
Haloperidol	37 (0.15)
Others	
Lorazepam	578 (2.39)
Hydroxyzine	1,441 (5.97)
Polypharmacy	
CNS stimulants + Alpha-2 agonist or SSRI or antipsychotic	3,077 (12.74)
CNS stimulants + Alpha-2 agonist + SSRI or antipsychotic	665 (2.75)
CNS stimulants + Alpha-2 agonist	2,887 (11.95)
CNS stimulants + SSRI	434 (1.80)
CNS stimulants + Antipsychotics	468 (1.94)
Psychotherapy	
Psychotherapy services and procedures	2,442 (10.11)

*Note.* SSRI = selective serotonin reuptake inhibitors.

### Medication Prescription Rates (Table 2)

Over one-third (37%) of the children diagnosed with ADHD were prescribed ADHD medications. Over one-fourth (26%) were prescribed CNS stimulants (MPH or amphetamine), and around 22% were prescribed alpha-2 agonists during the study period. The prescription of other ADHD medications (atomoxetine, viloxazine, and bupropion) was low (<1%). MPH preparations (21%) were the most frequently prescribed CNS stimulants, and guanfacine (16%) was the most frequent non-stimulant. Around 4% of children were prescribed antidepressant medications, and the most common antidepressants were sertraline and fluoxetine (both more than >1%). Approximately 3% were prescribed antipsychotics, most for risperidone (2.2%). Prescriptions of mood stabilizers were low (valproate 0.77%).

### Polypharmacy (Table 2)

Around 13% of children prescribed CNS stimulants were also prescribed another psychotropic medication (alpha-2 agonists or antidepressants or antipsychotics). The most common combination was a CNS stimulant with alpha-2 agonist (12%). Approximately 3% were prescribed 3+ psychotropic medications (CNS stimulants, alpha-2 agonists and antidepressants or antipsychotics).

### Medication Persistence (Table 3)

To assess treatment persistence, we examined the percentage of children who were in the database for at least 6 months over the assessment period and had ≥6 prescriptions. Around 63% met this criterion for MPH and 56% for amphetamine. For youth prescribed alpha-2 agonists, around 38% for clonidine and 34% for guanfacine had at least six or more prescriptions.

**Table 3.** Treatment Persistence.^a^

Medication CLass (total number of prescriptions)	= or >6 Code (%)
CNS stimulants	
Methylphenidate (N2348)	1,475 (62.82)
Amphetamines (N1175)	653 (55.57)
Alpha-2 agonists	
Clonidine (N1445)	555 (38.40)
Guanfacine (N1957)	658 (33.62)

a Analysis based on children who have at least 6 months of assessment periods in the cohort.

### Psychotherapy Services and Therapy Persistence

Psychotherapy services were much less frequent than pharmacological intervention as only one-tenth (*N* = 2,442) had any billing code for psychological services during their entire assessment period. Approximately 50% (*N* = 1,228) of children receiving therapy also used medication at some point with 14% of the sample ever accessing both ADHD medication and therapy during the assessment period, leaving 86% of medicated youth who never accessed therapy. Among those who had psychotherapy services and were in the database for at least 4 months (*N* = 1490), more than half (*N* = 805, 54.02%) had at least four visit codes related to psychotherapy.

### Treatment Sequences

Around 87% (*N* = 7807) were prescribed ADHD medications before any psychotherapy services. Among those ever-used therapy services, approximately two-thirds (*N* = 1,566, 64%) received psychotherapy before trying any ADHD medications. Almost all (*N* = 2,360, 97%) received psychotherapy before trying any antipsychotic medication. The use of other psychotropics typically came after the prescription of ADHD medications. For example, 95% (*N* = 8,493) of children on ADHD medication were first prescribed ADHD medication without prior antipsychotic medications trial. Similarly, 93% (*N* = 8,304) of youth on ADHD medication were first prescribed an ADHD medication without a previous antidepressant trial.

### Ethnicity, Race and Prescription Pattern (Table 4)

Hispanic youth were less likely to be prescribed ADHD medications (OR 0.80, 95% CI [0.74, 0.87]), CNS stimulants (OR 0.82, 95% CI [0.75, 0.90]), alpha-2 agonists (OR 0.71, 95% CI [0.64, 0.79]), or polypharmacy (OR 0.69, 95% CI [0.61, 0.78]), but received more therapy services (OR 1.26, 95% CI [1.12, 1.42]) versus non-Hispanic youth. There was no difference in the prescription patterns for antidepressants and antipsychotic medications between these two ethnic groups. White children were more likely than non-white children to be prescribed all classes of medications (ADHD, antidepressants, antipsychotics) and polypharmacy, as well as therapy services (OR 1.27, 95% CI [1.17, 1.38]).

**Table 4.** Race, Ethnicity, Comorbidity, and Prescription Pattern.

	Hispanic 2,982 (%)	Non-Hispanic 21,169 (%)	OR	95% CI
ADHD medication	968 (32.46)	7,954 (37.57)	0.80	[0.74, 0.87]
CNS stimulants	687 (23.04)	5,666 (26.77)	0.82	[0.75, 0.90]
Alpha-2 agonists	521 (17.47)	4,858 (22.95)	0.71	[0.64, 0.79]
Antidepressants	121 (4.06)	836 (3.95)	1.07	[0.88, 1.30]
Antipsychotics	94 (3.15)	641 (3.03)	1.04	[0.84, 130]
Polypharmacy (≥2)	287 (9.62)	2,829(13.36)	0.69	[0.61, 0.78]
Therapy services	359 (12.04)	2,079 (9.82)	1.26	[1.12, 1.42]
	White 12,902 (%)	Nonwhite 11,249 (%)		
ADHD medication	5,494 (42.58)	3,452 (30.69)	1.68	[1.59, 1.77]
CNS stimulants	3,944 (30.57)	2,409 (21.42)	1.62	[1.52, 1.71]
Alpha-2 agonists	3,331 (25.82)	2,048 (18.21)	1.56	[1.47, 1.66]
Antidepressants	628 (4.87)	308 (2.74)	1.87	[1.63, 2.15]
Antipsychotics	432 (3.35)	275 (2.44)	1.38	[1.19, 1.61]
Polypharmacy (≥2)	2,011 (15.59)	1,105 (9.82)	1.70	[1.57, 1.83]
Therapy services	1,431 (11.09)	1,007 (8.95)	1.27	[1.17, 1.38]
	ADHD with comorbidity^a^ 10,206 (%)	ADHD without comorbidity 13,945 (%)		
ADHD medication	4,665 (45.71)	4,281 (30.70)	1.90	[1.80, 2.00]
CNS stimulants	3,108 (31.45)	3,225(23.13)	1.46	[1.37, 1.54]
Guanfacine	2,350 (23.03)	1,409 (10.10)	2.66	[2.48, 2.86]
Clonidine	1,631 (15.98)	958 (6.87)	2.77	[2.55, 3.01]
Atomoxetine	109 (1.07)	97 (0.70)	1.54	[1.17, 2.03]
Antidepressants	745 (7.30)	192 (1.38)	5.64	[4.80, 6.62]
Antipsychotics	548 (5.37)	160 (1.15)	4.89	[4.09, 5.84]
Polypharmacy (≥2)	1,970 (19.30)	1,139 (8.17)	2.69	[2.49, 2.91]
Therapy services	1,636 (16.03)	803 (5.76)	3.12	[2.86, 3.41]
	ADHD with sleep disorders 6,227 (%)	ADHD without sleep disorders 17924 (%)		
ADHD medication	3,172 (50.94)	5,774 (32.21)	2.18	[2.06, 2.32]
CNS stimulants	1,952 (31.35)	4,401 (24.55)	1.40	[1.32, 1.49]
Guanfacine	1,581 (25.39)	2,185 (12.19)	2.45	[2.28, 2.64]
Clonidine	1,576 (25.31)	1,015 (5.66)	5.98	[5.14, 6.52]
Atomoxetine	62 (1.00)	144 (0.80)	1.24	[0.92, 1.67]
Antidepressants	494 (7.93)	447 (2.49)	3.37	[2.95, 3.84]
Trazodone	141 (2.26)	29 (0.16)	14.30	[9.58, 21.34]
Antipsychotics	307 (4.93)	402 (2.25)	2.26	[1.94, 2.63]
Hydroxyzine	726 (11.66)	715 (3.99)	3.18	[2.85, 3.54]
Polypharmacy (≥2)	1,366 (21.94)	1,750 (9.76)	2.88	[2.66, 3.11]
Therapy services	864 (13.88)	1,578 (8.80)	1.67	[1.53, 1.82]
	ADHD with seizure disorder 964 (%)	ADHD without seizure disorder 23,187 (%)		
ADHD medication	410 (42.53)	8,512 (36.12)	1.27	[1.12, 1.45]
CNS stimulants	210 (21.78)	6,143 (26.49)	0.77	[0.66, 0.90]
Alpha-2 agonists	309 (32.05)	5,070(21.87)	1.69	[1.47, 1.94]
Antidepressants	66 (6.85)	875 (3.77)	1.87	[1.45, 2.43]
Antipsychotics	74 (7.68)	635 (2.74)	3.04	[2.36, 3.90]
Therapy services	85 (8.82)	2,356 (10.16)	0.85	[0.68, 1.07]
	ADHD with low BMI^b^ 686(%)	ADHD without low BMI 23,465 (%)		
ADHD medication	283 (41.25)	8,639 (36.83)	1.21	[1.03, 1.41]
CNS stimulants	206 (30.03)	6,147 (26.20)	1.21	[1.02, 1.43]
Alpha-2 agonists	170 (24.78)	5,208 (22.19)	1.15	[0.97, 1.38]
Antidepressants	29 (4.23)	912 (3.89)	1.09	[0.75, 1.59]
Antipsychotics	29 (4.23)	680 (2.90)	1.54	[1.04, 2.23]
Therapy services	105 (15.31)	2,336 (9.96)	1.63	[1.32, 2.02]

*Note*. BMI = Body Mass Index.

a Comorbid Diagnoses: autism spectrum disorder or anxiety disorders or disruptive behavior disorders.

b BMI pediatric, = to <5 percentiles for age.

### Comorbidity and Prescription Pattern (Table 4)

All medication classes and therapy services were more likely in children with both ADHD and another behavioral health disorder (ASD or anxiety disorders or disruptive behavior disorders) versus youth without additional comorbidities, with the highest odd ratios for antidepressant (OR 5.64, 95% CI [4.80, 6.62]) and antipsychotic (OR 4.89, 95% CI [4.09, 5.84]) medications. Similarly, except for atomoxetine (OR 1.24, 95% CI [0.92, 1.62]), prescription rates of all medication groups and billable therapy services were much higher in children with ADHD and sleep disorders versus those without sleep disorders, with the highest odd ratios for trazodone (OR 14.30, 95% CI [9.58, 21.34]) and clonidine (OR 5.98, 95% CI [5.14, 6.52]).

CNS stimulants (OR 0.77, 95% CI [0.66, 0.90]) were prescribed less frequently among children with ADHD and seizure disorder, while other classes of medications (ORs range 1.69–3.04) were prescribed more likely than children without seizure disorder. There was no difference between therapy services between these two groups. Children with ADHD and low BMI were more likely to be prescribed ADHD medication (OR 1.21, 95% CI [1.03, 1.41]), CNS stimulants (OR 1.21, 95% CI [1.02, 1.43]), antipsychotics (OR 1.54, 95% CI [1.04, 2.23]) and therapy services (OR 1.63, 95% CI [1.32, 2.02]), but there was no difference in prescription pattern for alpha-2 agonists and antidepressants than children without low BMI.

### Pandemic and Treatment Pattern

**Figure 1.**
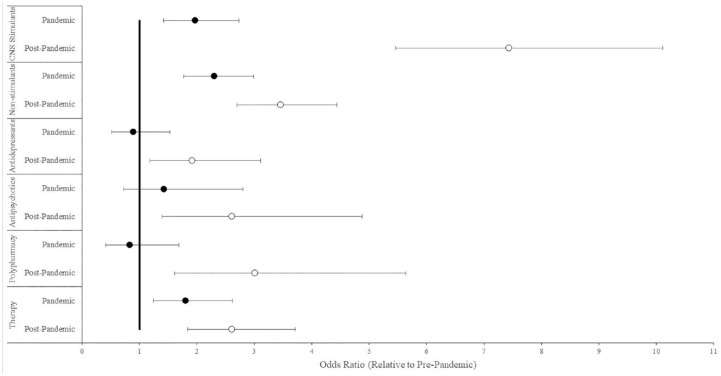
Pandemic and treatment pattern. *Pre-pandemic (time frame: Jan 2017–Feb 2020, *N* = 859); Pandemic (time frame: March 2020–July 2021, *N* = 3,511); Post-pandemic (time frame: August 2021 to August 2023, *N* = 21,763).

All medication classes and therapy services were more frequently utilized during pandemic and post-pandemic times than during pre-pandemic, except for antidepressants, antipsychotics, and polypharmacy during pandemic versus pre-pandemic periods (Figure 1). The highest odd ratio was for CNS stimulants (OR 7.43, 95% CI [5.46, 10.11]) during the post-pandemic period. There was an appreciable increase for all other treatments, including other medication classes (ORs 1.92–3.46) and therapy services (OR 2.61, 95% CI [1.84, 3.71]) in post-pandemic time.

## Discussion

The goal of this study was to identify treatment patterns for preschool-aged youth with ADHD to identify barriers and potential solutions to increasing access to evidence-based interventions and guideline-endorsed care (Pliszka & Aacap Work Group On Quality Issues, 2007; Wolraich et al., 2019). More than one-third of preschoolers with ADHD were prescribed ADHD medication, while only one-tenth had a billing code for any psychotherapy service over the entire assessment period. Prescription rates for CNS stimulants were much higher than alpha-2 agonists, with MPH and guanfacine being the most frequently prescribed agents within their classes. In contrast to what is recommended by care guidelines in the U.S. and Europe (NICE, 2018; Wolraich et al., 2019), most preschoolers were prescribed ADHD medications without evidence of prior psychotherapy. Polypharmacy rates were higher than psychotherapy rates with the most common combination of medications being a CNS stimulant and an alpha-2 agonist. Persistence prescription for CNS stimulants was much higher than therapy services and alpha-2 agonists. Prescriptions for other psychotropic medications including antipsychotics, antidepressants and mood stabilizers were low and tended to come after prescriptions for CNS stimulant medications for ADHD. Hispanic ethnicity was associated with increased odds to receive psychotherapy services. White race was associated with being more likely to be prescribed all classes of psychotropic medications, and nonwhite families were less likely to access all ADHD treatments including therapy. Prescriptions of antidepressants and antipsychotics were associated with comorbid psychiatric diagnoses and higher numbers of prescriptions of trazodone and clonidine were associated with sleep disorders. The impact of medical comorbidities was variable across medication classes and therapy utilization. Prescription of all classes of medications and therapy services increased significantly over the post-pandemic period as compared to pre-pandemic, with the highest increase in CNS stimulants prescriptions.

In this retrospective large cohort analysis, CNS stimulants were the most prescribed medications for preschoolers with ADHD. Rates of MPH were almost double that of amphetamine. This is consistent with AAP guidelines (Wolraich et al., 2019), which recommends MPH as the first-choice pharmacotherapy after psychosocial treatment because of the more robust evidence base for MPH in preschool children despite the lack of Food and Drug Administration (FDA) approval (Mattingly et al., 2021). Harstad and colleagues (Harstad et al., 2021) observed similarly low rates of amphetamine products, suggesting that when prescribers prefer a stimulant, they are consistent with AAP recommendations preferring MPH over amphetamine. This study and others have found an appreciable number of preschoolers with ADHD are prescribed alpha-2 agonists despite the lack of any controlled trials in this age range (Mittal et al., 2020). The overall ratio of stimulant to non-stimulant medications in preschool age youth prescribed by developmental pediatricians was comparable to what was observed here for a wide range of prescribers (Blum et al., 2018; Harstad et al., 2021). While TriNetX does not allow for coding of prescriber type, these combined results suggest that specialists have similar prescribing habits to generalists given that most ADHD medications in children are prescribed by primary care versus specialty pediatrics or child psychiatry (Sultan et al., 2018). Harstad and colleagues (Harstad et al., 2021) observed that use of alpha-2 agonists was more likely in children under 4 versus older than four and that they were the initial medication in over one-third of cases. Similarly, Davis and colleagues reported more prescription of alpha-2 agonists in children 2 to 4 years of age, while use of CNS stimulants in 5-year-old children, as well as increase in alpha-2 agonists use and decrease use of CNS stimulants from 2012 to 2017 (Davis et al., 2020). These results suggest that prescribers view this class of medication to be safe in very young children, or at least safer than CNS stimulants, despite the lack of controlled data on their tolerability. In older children, alpha-2 agonists are much more likely to be used with CNS stimulants than as monotherapy (Davis et al., 2020; Guevara et al., 2002). We were not able to ascertain the precise % of alpha-2 agonists used adjunctively but 12% of the sample had been prescribed them with CNS stimulants. It appears that prescribers are comfortable using these two medications together even in young children (Davis et al., 2020).

The AAP and NICE guidelines recommend behavior therapy services as the first line treatment for preschoolers with ADHD (NICE, 2018; Wolraich et al., 2019). It is encouraging that rates have increased 3-fold over the past 5 to 6 years as earlier studies failed to document any increase in services after the release of AAP guidelines in 2011 recommending behavioral services before medication (Visser et al., 2016; Waxmonsky et al., 2019). A recent study from outside of the U.S. also found that primary care clinicians typically discuss behavioral services as an option for all ages (Zysset et al., 2023). However, despite these gains over time, overall utilization rates were low. Even with increases seen over the pandemic, therapy rates did not surpass 10%. This rate is lower than what has been reported elsewhere for preschoolers and older children where rates ranged between 30% and 63% (Danielson, Bohm et al., 2023; Danielson, Visser et al., 2018; Visser et al., 2014), likely in part due to TriNetx’s inability to capture school or center-based services such as Head Start that are not billed to insurance. However, the majority of these services target academic versus behavioral functioning and are preferentially offered to preschoolers with ADHD with a comorbid learning disability or other neurodevelopmental delays (DuPaul et al., 2019). Moreover, the frequency of school-based psychosocial support has decreased over the pandemic, placing greater priority on the insurance-based services assessed in this study (Danielson, Claussen et al., 2023). There is also a possibility that a number of patients referred for therapy services might have received treatment from community providers outside of the host healthcare network that TriNetX could not capture. In addition, variances in sample composition may have impacted rates.

Over time, use of ADHD medications has increased in children as 6.1% of children ages 4 to 17 years were prescribed medication in 2011 as compared to 4.8% of youth with ADHD in 2007 (Visser et al., 2014). In 2016, around 18% of parents of preschool-aged U.S. children reported using ADHD medication alone or in combination with therapy (Danielson, Bitsko et al., 2018), while there was slight decrease in prescription of stimulants for in children ages 5 and above from 2016 to 2021 (Danielson, Bohm et al., 2023). In contrast to this pre-pandemic decline in treatment rates, we observed a doubling of ADHD prescriptions and therapy services during the first year of the pandemic (Spring 2020 until summer 2021). Similarly, others have also reported large single-year increases in CNS stimulant prescription rates during the first year of the pandemic (2020–2021) for adolescents and adults compared to the average annual % change between 2016 and 2020 (Danielson, Bohm et al., 2023). In contrast to ADHD medications, we observed either a decrease or no change for other psychotropic medications in preschoolers during the first year of the pandemic. Over the entire assessment period which spanned into 2023, all prescriptions of all medications increased even for those agents with an initial decline in use. However, there was an appreciably greater increase in CNS stimulants from the summer of 2021 onward than for all other treatments, with a 7-fold increase in the past 2 years as compared to prior to the pandemic. While this is the first study to examine trends specifically in preschool youth, others have observed that children (ages 5–11) experienced larger increases in psychotropic medication usage during the pandemic than adolescents or adults (Bliddal et al., 2023). In Finland, rates of psychotropic medication in youth rose by approximately 50% during the second year of the pandemic when in-person schooling was more common (Kuitunen, 2022). However, the increase was much less than what was observed here in the U.S. and confined only to CNS stimulants, whereas we observed around tripling in all measured medication classes with the exception of a milder 2-fold increase in antidepressants. Studies of adults in the U.S. also found an increase across most measured classes of psychotropics versus just being confined to CNS stimulants (Bliddal et al., 2023).

Rates of polypharmacy exceeded therapy usage in our sample suggesting that prescribers and/or parents may be more apt to start a second medication before engaging in therapy services, which is inconsistent with all published care guidelines for ADHD care in these ages. There is a larger database for multimodal ADHD care than there is for polypharmacy trials of ADHD across all age ranges. Adding behavioral therapy to a single ADHD medication has been found to improve outcomes across different domains of functioning and reduce the medication dose needed to achieve optimal outcomes (Arnold et al., 2015; Pelham et al., 2014, 2016).

The increased burden of mental health conditions during COVID-19 on parents and children may explain the national trend of increasing psychotropic usage across ages. In the U.S., many school-aged children participated in low-intensity remote learning during the first year of the pandemic and then returned to in-person school in the fall 2021–22 school year after the release of the “Return to School Roadmap” by the U.S. Department of Education (U.S. Department of Education, 2021). This transition in school settings and associated increase in academic demands may explain the large rise in ADHD medication usage during the later years of the pandemic. The numerous policy changes in mental health treatment both at the federal and state level, including leniency around prescriptions of controlled drugs via telehealth, may have led to preferential increase in CNS stimulants which are the only controlled substance medication class that we tracked in this analysis.

This data provides some support that pandemic-inspired healthcare policy changes increased utilization of a wide range of behavioral health services for children. These include increased reimbursement and coverage for telehealth services and home delivery options for patients. For example, recent studies have also shown that tele-visits were associated with improved attendance rates (Kris, 2023; Muppavarapu et al., 2022). Given the appreciable challenges connecting families with therapy services for their children, extension of these access-improving policies should be considered regardless of the status of the pandemic. Despite these access gains, the increase in therapy services over the pandemic was only one-third of that seen for CNS stimulant medication, comparable to that seen for antipsychotic medications in this age range, and less than the increase in rates of psychotropic polypharmacy. The differential rate of increase in medication prescribing versus therapy services over the pandemic provides further evidence that provider availability is not the only barrier. As initial use of medication treatments for ADHD has been shown to inhibit future uptake of therapy, this widening gap may predict even further challenges increasing therapy utilization rates in the future (Pelham et al., 2016; Waxmonsky et al., 2019).

Utilization of antipsychotics were low and typically preceded by a trial of an established ADHD medication. When antipsychotics were prescribed in this age range, ADHD is the primary diagnosis in this sample as well as in others (Chen et al., 2021; Kamble et al., 2015; Vitiello et al., 2015) suggesting that a diagnosis of ADHD at an early age is an appreciable risk factor for future antipsychotics prescription. The rates observed here are 3 to 4-fold less than in older youth with ADHD (Girand et al., 2020) possibly due to the higher rates of comorbidity as well as the greater comfort of specialists with these medication classes in older versus younger youth. After a decade of increasing rates of antipsychotics in youth, their use has leveled off in the past 10 years (Mackie et al., 2021). In addition, the rates of therapy before antipsychotics are much improved versus earlier studies which found under half of children first accessed therapy (Finnerty et al., 2016). However, these positive trends appeared to reverse during the coronavirus pandemic with a 2.6-fold increase of antipsychotics in the last 2 years. Moreover, it was concerning to see that the rate of increase in antipsychotics was identical to that for therapy over the course of the pandemic.

ADHD is a chronic health condition that often requires extended treatment durations for optimal outcomes (Boland et al., 2020). Past work has shown that the degree of medication adherence to CNS stimulants is associated with improved outcomes (Baweja, Waschbusch, Pelham, et al., 2021; Biederman et al., 2009). Numerous studies show that upwards of 50% of patients stop ADHD medications within 6 to 12 months of initiation (Baweja, Soutullo, & Waxmonsky, 2021; Marcus et al., 2005; Raman et al., 2015). In this sample, between 56% and 63% of patients were prescribed six or more scripts for CNS stimulants during the assessment period. These numbers may be higher than what was reported elsewhere given our coarse measure of adherence based on prescriptions dispensed in the medical record and the variable duration of the assessment period (6–66 months). Patients were less likely to fill six or more prescriptions for non-stimulants (under 39% rate) than for CNS stimulants. A retrospective claims study of youth and adults also found adherence to medications was greater for CNS stimulants in comparison to nonstimulant medications (Christensen et al., 2010). The shortened duration of use with nonstimulants could reflect issues with cost, efficacy or tolerability. Even though alpha-2 agonists are advised to be taken daily for optimal efficacy and tolerability, as needed dosing for sleep problems is not uncommon (Ming et al., 2008) and could also explain the relatively low rate of script refills for this medication class. Prior work has documented that therapy services for children with ADHD often end prematurely (Kazdin, 1996). In youth 17 years and under, just over 50% had four billable sessions in any year (Waxmonsky et al., 2019), which is considered a minimally effective dose (Fabiano et al., 2009). Similar rates were seen in this sample, highlighting the barriers families appear to face for initiating and persisting with therapy services for ADHD. These challenges are not limited to areas with few behavioral health resources, as increased provider density does not appreciably improve therapy utilization rates for children (Gellad et al., 2014).

Treatment of children and adolescents for ADHD in the U.S. has been found to vary by child age, race, ethnicity, socioeconomic status, and health insurance status but not gender (Danielson, Bitsko et al., 2018). Younger age at diagnosis is associated with increased utilization of the spectrum of ADHD services, suggesting the value of early detection. Regarding race and ethnicity, medication use is higher in white versus nonwhite populations and therapy rates higher in public versus private insured families (Baweja, Soutullo, & Waxmonsky, 2021). We observed similar trends here for ADHD medications with youth of Hispanic ethnicity being less likely to be prescribed ADHD medications and any polypharmacy combination than patients of non-Hispanic ethnicity. Rates of therapy were higher in those of Hispanic ethnicity. In contrast, non-white youth had lower rates of all treatments than white patients, including therapy. The appreciable impact of race and ethnicity on ADHD care may be related to cultural perceptions toward ADHD and specifically about medication use for ADHD (Baweja, Soutullo, & Waxmonsky, 2021). The data supports a more global access barrier for non-white families. While it was encouraging to see that Hispanic ethnicity was a positive predictor of therapy services, barely one in 10 children of Hispanic youth had a single billable therapy code suggesting substantive progress in access to therapy is needed for all children regardless of race or ethnicity. We were not able to assess associations with insurance (public vs. private) or prescriber type (psychiatry vs. primary care) in the TriNetX database, which could confound observed associations with ethnicity or race and explain lower rates of therapy seen in non-white patients than reported elsewhere (Danielson, Bitsko et al., 2018).

There are well established associations between psychiatric comorbidity and treatment patterns in children and adults with ADHD (Baweja & Waxmonsky, 2018, 2022; Faraone, 2018; Girand et al., 2020). A number of factors have been associated with polypharmacy rates in preschoolers (Girand et al., 2020) ranging from demographics and geography to insurance type and uptake of counseling services. In this sample, preschoolers with ADHD and associated comorbidities were more likely to receive all medication classes including polypharmacy as well as therapy services. It appears that providers rely on the presence of symptoms not traditionally associated with ADHD as an indicator to use alternate medication classes or to refer to therapy. However, the greatest increases associated with psychiatric comorbidity were for antidepressants and antipsychotics. These increases were over 50% larger than that seen for therapy services, despite these two medication classes having smaller evidence bases than therapy for this age range.

ADHD is commonly associated with sleep problems in children, adolescents and adults (Ogundele & Yemula, 2022). It remains unclear how much of these associations are due to the direct effects of ADHD versus the potential adverse effects of CNS stimulants on sleep. The presence of comorbid sleep problems was one of the most robust predictors of treatment patterns with all services more likely in youth with concurrent sleep problems except for atomoxetine. The initial management of sleep disorders in children with ADHD includes sleep hygiene promotion and other behavioral interventions (Cortese et al., 2013). There is a more robust evidence base for nonpharmacological treatments of insomnia in children than there is for pharmacological treatments (Dewald-Kaufmann et al., 2019; Lunsford-Avery et al., 2021). The presence of sleep disorders was associated with 1.67 increased odds of therapy, but this increase was smaller than for all assessed medication classes other than CNS stimulants. There is only limited evidence to support the use of alpha-2 agonists such as clonidine to improve sleep onset latency in individuals with ADHD (Prince et al., 1996; Wilens et al., 1994) and even less data to support the use of trazodone (Bruni et al., 2018). Clonidine prescription rate increased over 6-fold and trazodone 14-fold in youth with sleep problems versus those without. These findings parallel what is seen in older youth where alpha-2 agonists were the most prescribed insomnia medication for 81% of the children with ADHD (Owens et al., 2010). Use of hydroxyzine was also more common in preschoolers with ADHD and sleep problems, consistent with trends observed in youth without ADHD who have sleep problems, where hydroxyzine and diphenhydramine are the most prescribed interventions (Bruni et al., 2018). The preferential avoidance of atomoxetine versus stimulant medication is somewhat surprising given a study showing a more favorable sleep profile for the former than the latter (Sangal et al., 2006).

Overall, the impact of medical comorbidities was less than psychiatric comorbidities or sleep problems. Seizure disorder was the only comorbidity associated with reduced use of CNS stimulants in these ages that are prone to have more challenges tolerating these agents than older patients (Wolraich et al., 2019). The lower utilization is not surprising given the potential for these agents to impact the seizure threshold, especially when seizures are not well controlled (Man et al., 2020). Providers appeared to preferentially use alpha-2 agonists versus therapy to fill the treatment void, despite the very limited safety data for this medication class in patients with seizures (Andrade, 2020; Torres et al., 2008). While the numbers are small, it was surprising to see a 3-fold increase in antipsychotic use in preschoolers with versus without seizure disorders as these agents also impact the seizure threshold and have a much smaller evidence base than CNS stimulants. In contrast to seizures, low BMI was associated with increased use of CNS stimulants even though appetite loss and growth suppression are appreciably more likely to occur with CNS stimulants than nonstimulant medications (Baweja, Hale, & Waxmonsky, 2021). Unlike seizures, therapy services were more likely in those with low BMI.

This multicentric retrospective study has several limitations, the primary of which was not being able to capture treatment information from settings that do not participate in TriNetX, for example, Head Start and other school services. Unfortunately, there are few datasets in the United States which offer the depth of treatment level data seen in TriNetX in a broader more representative sample which would capture the plethora of settings where children receive behavioral health services. Until such data is available, we are forced to integrate findings across different studies and samples. Additional limitations are also inherent to the TriNetX, including lack of structured measures of efficacy or tolerability as well as information about insurance, prescriber types and socioeconomic status. This sample size of TriNetX searches varies with the search date as the data set is continuously adding new members. Therefore, our analysis had appreciably more cases in the post-pandemic period compared with the pre-pandemic period. We also used a rough measure of adherence defined as frequency of prescription dispensed which does not account for what families do with the medication once it is prescribed. Regarding polypharmacy, the nature of this dataset limits the ability to differentiate if medications were used at the same time, or if one replaced another. Approximately one-fourth of the sample was missing information on race and ethnicity, which might have impacted the results of the sub-analysis of these demographic variables on treatment trends. As the database collects only limited demographic information and its sample is dependent on which health systems enroll, it is unclear how representative these findings are for national trends. Still, this study represents one of the largest samples of preschoolers with ADHD to date.

## Conclusions

In this large electronic health record analysis of nearly 24,000 children ages 3 to 6 years diagnosed with ADHD, one in three were prescribed medication for ADHD, most commonly methylphenidate or guanfacine. Only one in 10 accessed psychotherapy services that could be identified through their healthcare records. Results were largely inconsistent with guideline-recommended care as ADHD medication preceded therapy and a wide range of medications were prescribed beyond methylphenidate. Rates of all treatments increased over the course of the coronavirus pandemic, with CNS stimulant usage accelerating the most. Psychiatric comorbidity and sleep problems appreciably increased rates of all treatment services with a less robust impact of medical comorbidities and demographic features. These data suggest the need for further efforts to promote care consistent with established guidelines.

## Supplemental Material

sj-docx-1-jad-10.1177_10870547231215287 – Supplemental material for Treatment Utilization Pattern of Preschool Children With Attention-Deficit/Hyperactivity Disorder Supplemental material, sj-docx-1-jad-10.1177_10870547231215287 for Treatment Utilization Pattern of Preschool Children With Attention-Deficit/Hyperactivity Disorder by Raman Baweja, Ritika Baweja, Hunter Weidlich, Jennifer E. Nyland, Daniel A. Waschbusch and James G. Waxmonsky in Journal of Attention Disorders
