# Supplementary material for: Treatment Utilization Pattern of Preschool Children With Attention-Deficit/Hyperactivity Disorder
Source: J Atten Disord. 2023 Dec 12;28(5):708–21. doi: 10.1177/10870547231215287 (PMC10928959; doi:10.1177/10870547231215287)
Supplement: sj-docx-1-jad-10.1177_10870547231215287 – Supplemental material for Treatment Utilization Pattern of Preschool Children With Attention-Deficit/Hyperactivity Disorder [file sj-docx-1-jad-10.1177_10870547231215287.docx]

**Appendix**

**DSM-5 code**

| F90 | Attention-deficit hyperactivity disorders |
| --- | --- |
| F84 | Autism Spectrum Disorder |
| F91 | Disruptive behavior disorders |
| F91.3 | Oppositional defiant disorder |
| F91.1 | Conduct disorder, childhood-onset |
| F34.8 | Disruptive mood dysregulation disorder |
| F32 | Depressive episode (Including MDD) |
| F31 | Bipolar disorder |
| F41.9 | Anxiety disorder, unspecified |
| F41.1 | Generalized anxiety disorder |
| F93.0 | Separation anxiety disorder of childhood |
| F43.1 | Post-traumatic stress disorder |
| F70-79 | Intellectual Disabilities |
| F95 | Tic disorder |
| G47 | Sleep Disorders |
| F50 | Eating Disorders |
| F80 | Specific developmental disorders of speech and Language |
| Z68.51 | BMI pediatric, < or = to 5 percentiles for age |
| G40 | Epilepsy and recurrent seizures |
| 90832-34, 36-40, 90846-7, 90849, 90853, 90785, 90882, 90885, 90887 | Psychotherapy Services and Procedures |
